# Complete mitochondrial genome of *Brochis multiradiatus*

**DOI:** 10.1080/23802359.2019.1711227

**Published:** 2020-01-16

**Authors:** Baohong Xu, Hang Su, Qiaolin Liu, Ligang Lv, Kaijian Chen, Tiaoyi Xiao

**Affiliations:** a Hunan Engineering Technology Research Center of Featured Aquatic Resources Utilization, Hunan Agricultural University, Changsha, Hunan, P. R. China;; b Collaborative Innovation Center for Efficient and Health Production of Fisheries in Hunan Province, Changde, Hunan, P. R. China

**Keywords:** Complete mitochondrial genome, *Brochis multiradiatus*, fish

## Abstract

We reported the complete mitochondrial genome yielded by next-generation sequencing of *Brochis multiradiatus* in this study. The total length of the mitochondrial genome is 16,916 bp, with the base composition of 32.49% A, 25.47% T, 27.12% C, and 14.91% G. It contains 2 ribosomal RNA genes, 13 protein-coding genes, 22 transfer RNA genes, and a major non-coding control region (D-loop region). The arrangement of these genes is the same as that found in the Corydoras. The complete mitochondrial genomes of *B. multiradiatus* and other 12 species from Siluriformes were used for phylogenetic analysis using neighbor-joining method. The topology demonstrated that all species belong to four genera and are divided into two groups (Siluridae and Callichthyidae), the *B. multiradiatus* was clustered with genus Corydoras. *Brochis multiradiatus’* molecular classification is consistent with the external morphological feature results, so the information of the mitogenome could be used for future identification of *Brochis*.

*Brochis multiradiatus* belongs to Teleostei, Siluriformes, Callichthyidae, subfamily Corydoradinae. *Brochis multiradiatus* is common in Chinese ornamental fish market. Because there are two small ‘whiskers’ beside its mouth, which looks like a small mouse swimming in the water, it is called mousefish. Mousefish is a category fish just named from appearance, such as *Corydoras nattereri voucher* (Moreira et al. 2016), *Corydoras panda* (Liu, Liu, Xiao, et al. 2019), *Corydoras duplicareus* (Liu, Xu, Xiao 2019), *Corydoras arcuatus* (Liu, Xu, Xiao, Liu 2019), and *Corydoras sterbai* (Liu, Liu, Xu, et al. 2019), they are all named mousefish. In order to distinguish and identify *B. multiradiatus* in genome, we determined the mitochondrial genome sequence of the *B. multiradiatus* and carried out mitochondrial genome structure and phylogenetic analysis. The live sample of *B. multiradiatus* was collected from the Red Star Ornamental Fish Market in Changsha, Hunan Province, China (113.03E, 28.09 N). After anesthesia with MS-222 (3-Aminobenzoic acid ethyl ester methanesulfonate), dorsal muscle tissue was collected and preserved in 99% ethanol in Museum of Hunan Agricultural University. After DNA extraction (Tissue DNA Kit D3396-02, Omega, Bio-Tek, Norcross, GA) and sequencing library construction (Sangon Biotech, Shanghai, China), paired-end reads were sequenced using HiSeq XTen PE 150 of Illumina. After extracting the DNA of the tissue (Number: QT001), the sequence library was constructed. BBduk and BLASTp were used to assess and monitor data quality. NOVOPlasty and SPAdes were used for *de novo* assembly. MITOS2 server and Geneious R11 (Tan et al. 2019) were used to predict and annotate the mitochondrial genome. Geneious Tree Builder was used for phylogenetic analysis and building phylogenetic tree.

Totally 27,191,198 high-quality clean reads (150 bp PE read length) were obtained. The total length of the *B. multiradiatus* mitochondrial genome is 16,916 bp (GenBank accession number: MN641874), with the base composition of 32.49% A, 25.47% T, 27.12% C, and 14.91% G. It contains 2 ribosomal RNA genes, 13 protein-coding genes, 22 transfer RNA genes, and a major non-coding control region 1298 bp in length. The arrangement of these genes is the same as that found in the Siluriformes (Liu, Xu, Xiao 2019). All the protein initiation codons are ATG, except for *cox1* that begins with GTG. The complete mitogenomes of *B. multiradiatus* and other 12 species from Siluriformes were used for phylogenetic analysis. The neighbor-joining tree was built by Geneious with Tamura–Nei (genetic distance) model and global aligment with free end gaps (aligment type) showed all species belong to 4 genera are divided into two groups (Siluridae and Callichthyidae), and the *B. multiradiatus* was clustered with other species from genus Corydoras (Figure 1). *Brochis multiradiatus’* external morphological feature classification is consistent with the molecular classification results, so the information of the mitogenome could be used for future phylogenetic analysis and identification of *B. multiradiatus*.

**Figure 1.**
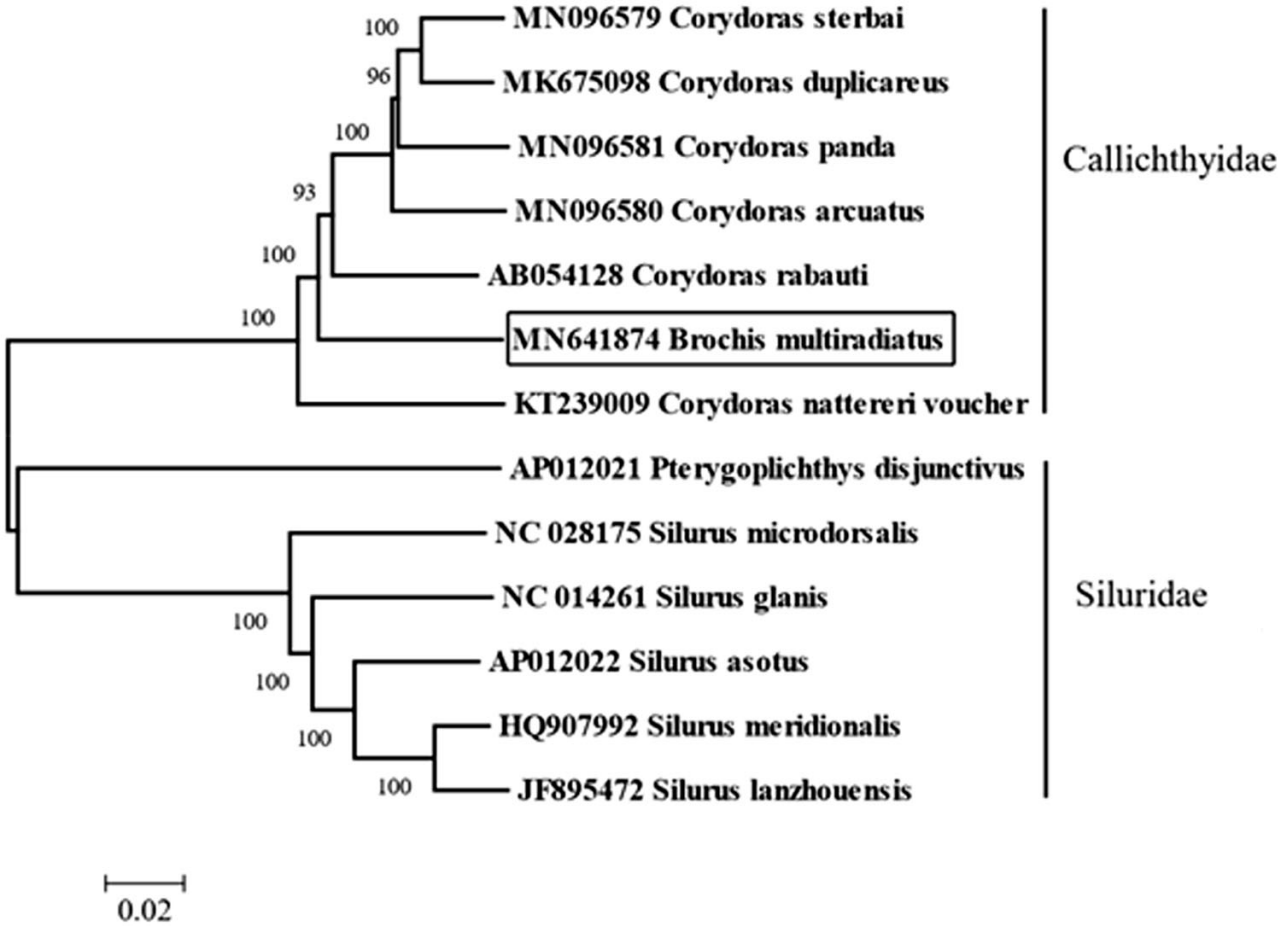
Neighbor-joining phylogenetic tree based on the complete mitochondrial genome sequence. The bold Latin name represents the species in this study. The codes following the Latin names were GenBank accession numbers for each mitogenome.
